# The role of primary health care in long-term care facilities during the COVID-19 pandemic in 30 European countries: a retrospective descriptive study (Eurodata study)

**DOI:** 10.1017/S1463423623000312

**Published:** 2023-10-24

**Authors:** Marina Guisado-Clavero, Sara Ares-Blanco, Alice Serafini, Lourdes Ramos Del Rio, Ileana Gefaell Larrondo, Louise Fitzgerald, Shlomo Vinker, Gijs van Pottebergh, Kirsi Valtonen, Bert Vaes, Canan Tuz Yilmaz, Péter Torzsa, Paula Tilli, Theresa Sentker, Bohumil Seifert, Natalija Saurek-Aleksandrovska, Martin Sattler, Goranka Petricek, Ferdinando Petrazzuoli, Davorina Petek, Ábel Perjés, Naldy Parodi López, Ana Luisa Neves, Liubovė Murauskienė, Heidrun Lingner, Katarzyna Nessler, Bruno Heleno, Anna Krztoń-Królewiecka, Milena Kostić, Büsra Çimen Korkmaz, Snežana Knežević, Aleksandar Kirkovski, Vasilis Trifon Karathanos, Marijana Jandrić-Kočić, Shushman Ivanna, Оксана Ільков, Kathryn Hoffmann, Miroslav Hanževački, Mila Gómez-Johansson, Dragan Gjorgjievski, Philippe-Richard J. Domeyer, Maryher Delphin Peña, Asja Ćosić Divjak, Iliana-Carmen Busneag, Elena Brutskaya-Stempkovskaya, Sabine Bayen, Maria Bakola, Limor Adler, Radost Assenova, María Pilar Astier-Peña, Raquel Gómez Bravo

**Affiliations:** 1 Investigation Support Multidisciplinary Unit for Primary Health Care and Community North Area of Madrid, Madrid, Spain; 2 Federica Montseny Health Centre, Gerencia Asistencial Atención Primaria, Servicio Madrileño de Salud, Madrid, Spain; Instituto de Investigación Sanitaria Gregorio Marañón, Madrid, Spain; 3 Azienda Unità Sanitaria Locale di Modena; Laboratorio EduCare, University of Modena and Reggio Emilia, Italy; 4 Federica Montseny Health Centre, Gerencia Asistencial de Atención Primaria, Servicio Madrileño de Salud, Madrid, Spain; 5 Member of Irish College of General Practice (MICGP), Member of Royal College of Physician (MRCSI), Ireland; 6 Department of Family Medicine, Sackler Faculty of Medicine, Tel Aviv University, Tel Aviv, Israel; WONCA Europe President; 7 Department of Public Health and Primary Health Care, KU Leuven, Leuven, Belgium; 8 Communicable Diseases and Infection Control Unit, City of Vantaa and University of Helsinki, Helsinki, Finland; 9 Department of Public Health and Primary Health Care, KU Leuven, Leuven, Belgium; 10 Lecturer, Bursa Uludağ University, Family Medicine Department, Turkey; 11 Department of Family Medicine, Semmelweis University, Hungary; 12 Communicable Diseases and Infection Control Unit, City of Vantaa and University of Helsinki, Helsinki, Finland; 13 Medizinische Hochschule Hannover, Hannover, Germany; 14 Charles University, First Faculty of Medicine, Institute of General Practice, Czech Republic; 15 Natalija, North Macedonia; 16 European Parliament, Luxembourg; 17 Department of Family Medicine “Andrija Stampar” School of Public Health, School of Medicine, University of Zagreb, Croatia; Health Centre Zagreb West, Croatia; 18 Department of Clinical Sciences in Malmö, Centre for Primary Health Care Research, Lund University, Malmö, Sweden; 19 Department of Family Medicine, Faculty of Medicine, University of Ljubljana, Slovenia; Chairperson of EGPRN; 20 Department of Family Medicine, University of Semmelweis, Budapest, Hungary; 21 Närhälsan Kungshöjd Health Centre, Gothenburg, Sweden; Department of Pharmacology, Sahlgrenska Academy, University of Gothenburg, Gothenburg, Sweden; 22 Imperial College London, United Kingdom; Faculty of Medicine, University of Porto, Portugal; 23 Department of Public Health, Institute of Health Sciences, Faculty of Medicine, Vilnius University, Lithuania; 24 Medizinische Hochschule Hannover, OE 5430, Carl Neuberg Str. 1, 30625 Hannover, Germany; 25 Department of Family Medicine, UJCM at Uniwersytet Jagielloński – Collegium Medicum, Poland; 26 Comprehensive Health Research Center, NOVA Medical School, Universidade Nova de Lisboa; USF das Conchas, Regional Health Administration Lisbon and Tagus Valley, Lisbon, Portugal; 27 Department of Family Medicine, Andrzej Frycz Modrzewski Krakow University, Krakow, Poland; 28 Health Center “Dr Đorđe Kovačević”, Lazarevac, Belgrade, Serbia; 29 Van Gürpınar District Public Hospital, Turkey; 30 Health Center Kraljevo, Kraljevo, Serbia; 31 Faculty of Medicine, Ss. Cyril and Methodius University, Skopje, North Macedonia; 32 Laboratory of Hygiene and Epidemiology, Medical Department, Faculty of Health Sciences, University of Ioannina-Greece; Family Doctor, GHS, Larnaca, Cyprus; 33 Health Center Krupa na Uni, Republic of Srpska, Bosnia and Herzegovina; 34 Department of Family Medicine and Outpatient Care, UZHNU, Medical Faculty 2, Ukraine; 35 Department of Family Medicine and Outpatient Care, Medical Faculty 2, Uzhhorod National University, Ukraine; 36 Associate Professor and Medical Doctor for General Practice and Primary Care, Medical University of Vienna, Austria; 37 Närhälsan Sannegården Health Centre, Gothenburg, Sweden; 38 Center for Family Medicine, Medical Faculty Skopje, North Macedonia; 39 School of Social Sciences, Hellenic Open University, Patras, Greece; 40 Department of Geriatric Medicine, Hôpitaux Robert Schuman, Luxembourg; 41 Health Centre Zagreb Centar, Zagreb, Croatia; 42 “Spiru Haret” University, Practising Family Doctor, Occupational Health Expert, Bucharest, Romania; 43 General Medicine Department, Belarusian State Medical University, Belarus; 44 Department of General Practice, University of Lille, UFR3S, France; 45 Research Unit for General Medicine and Primary Health Care, Faculty of Medicine, School of Health Science, University of Ioannina, Ioannina, Greece; 46 Department of Family Medicine, Sackler Faculty of Medicine, Tel Aviv University, Tel Aviv, Israel; 47 Department Urology and General Practice, Faculty of Medicine, Medical University of Plovdiv, Bulgaria; 48 Healthcare Quality Technical Assistant, Territorial Quality Unit, Camp de Tarragona Healthcare Directorate, Catalan Institute of Health, Catalonia Government, Spain; Semfyc, Wonca World Executive Board, University of Zaragoza, GIBA IIS Aragon, Spain; 49 Centre Hospitalier Neuro-Psychiatrique, CHNP, Rehaklinik, Ettelbruck, Luxembourg; 50 Research Group Self-Regulation and Health; Institute for Health and Behaviour, Department of Behavioural and Cognitive Sciences, Faculty of Humanities, Education, and Social Sciences, Luxembourg University, Luxembourg

**Keywords:** COVID-19, long-term care facility, nursing home, primary health care

## Abstract

**Background and aim:**

Primary health care (PHC) supported long-term care facilities (LTCFs) in attending COVID-19 patients. The aim of this study is to describe the role of PHC in LTCFs in Europe during the early phase of the pandemic.

**Methods:**

Retrospective descriptive study from 30 European countries using data from September 2020 collected with an ad hoc semi-structured questionnaire. Related variables are SARS-CoV-2 testing, contact tracing, follow-up, additional testing, and patient care.

**Results:**

Twenty-six out of the 30 European countries had PHC involvement in LTCFs during the COVID-19 pandemic. PHC participated in initial medical care in 22 countries, while, in 15, PHC was responsible for SARS-CoV-2 test along with other institutions. Supervision of individuals in isolation was carried out mostly by LTCF staff, but physical examination or symptom’s follow-up was performed mainly by PHC.

**Conclusion:**

PHC has participated in COVID-19 pandemic assistance in LTCFs in coordination with LTCF staff, public health officers, and hospitals.

## Introduction

There are more than 90 million people who are over 65 years old living in Europe (European Union, 2020). While most of them live in private households, some choose freely to move into long-term care facilities (LTCFs) or are forced to because they need more support due to their frailty. In 2018, there were 156,316 beds in LTCFs in Europe. The highest ratios of beds per 100,000 inhabitants were recorded in the Netherlands and in Sweden and the lowest in Greece and Bulgaria (Eurostat, 2022). The estimated number of LTCFs in the European Economic Area was calculated as 43,000 in December 2019 (ECDC, 2021).

COVID-19 has heavily impacted the oldest population, with 58% of COVID-19-related deaths in those over the age of 80 (Rocard *et al*., 2021). The estimated percentage of COVID-19 mortality in LTCFs in Europe was reported as ranging from 21 to 66% during the pandemic (Miralles *et al.*, 2021). It is worth nothing that before the pandemic, LTCFs faced difficulties in securing funding and coordinating properly with health systems due to shortage of staff (World Health Organisation, 2020). Additionally, health care support for LTCFs may vary depending on the type of health care provider from primary health care (PHC) professionals to geriatricians or hospital units’ teams and the organization of the health care system (Panza *et al.*, 2018). The World Health Organization (WHO) described PHC as a whole-of-society approach to effectively organize and strengthen national health systems to bring services for health and well-being closer to communities. PHC provision is organized in Europe under different health systems models (Böhm *et al.*, 2013) (Table 1).

**Table 1.** Different European models of health systems regarding regulation, financing, and provision (Organization for Economic Co-operation and Development)

Health system models	Regulation, financing, and provision	European countries
National Health System	State regulation, state financing, and state provision	Denmark, Finland, Island, Norway, Sweden, United Kingdom, Portugal and Spain
National Health Insurance	State regulation, state financing, and private provision	Irlanda and Italia
Social Health Insurance	Societal actors’ regulation, societal actors’ financing, and private provision	Austria, Germany, Luxembourg, Slovenia, Switzerland
Etatist Social Health Insurance	State regulation, societal actors’ financing, and private provision	Belgium, Estonia, France, Czech Republic, Hungary, Netherlands, Poland, Slovakia, Israel

Adapted from Böhm *et al*. (2013).

In Europe, the role of PHC in providing medical care to LTCFs varies considerably (Boeckxstaens and De Graaf, 2011). According to a study in seven high-income European countries, comprehensive care ranged 14–46% between LTCFs and general practitioners (GPs) (Doty *et al.*, 2020). At the beginning of the pandemic, the clinical pathways for COVID-19 patients in LTCFs were scarcely organized (Giri *et al*., 2021), although the WHO recommended early recognition and close monitoring of symptoms in residents and caregivers (WHO, 2022). The role of GPs in LTCFs during the pandemic has been poorly described and documented (Dykgraaf *et al.*, 2021). The aim of this study is to analyze the role of PHC in the clinical pathways for LTCF COVID-19 patients in 30 European countries in the early phase of the pandemic.

## Methods

**Figure 1.**
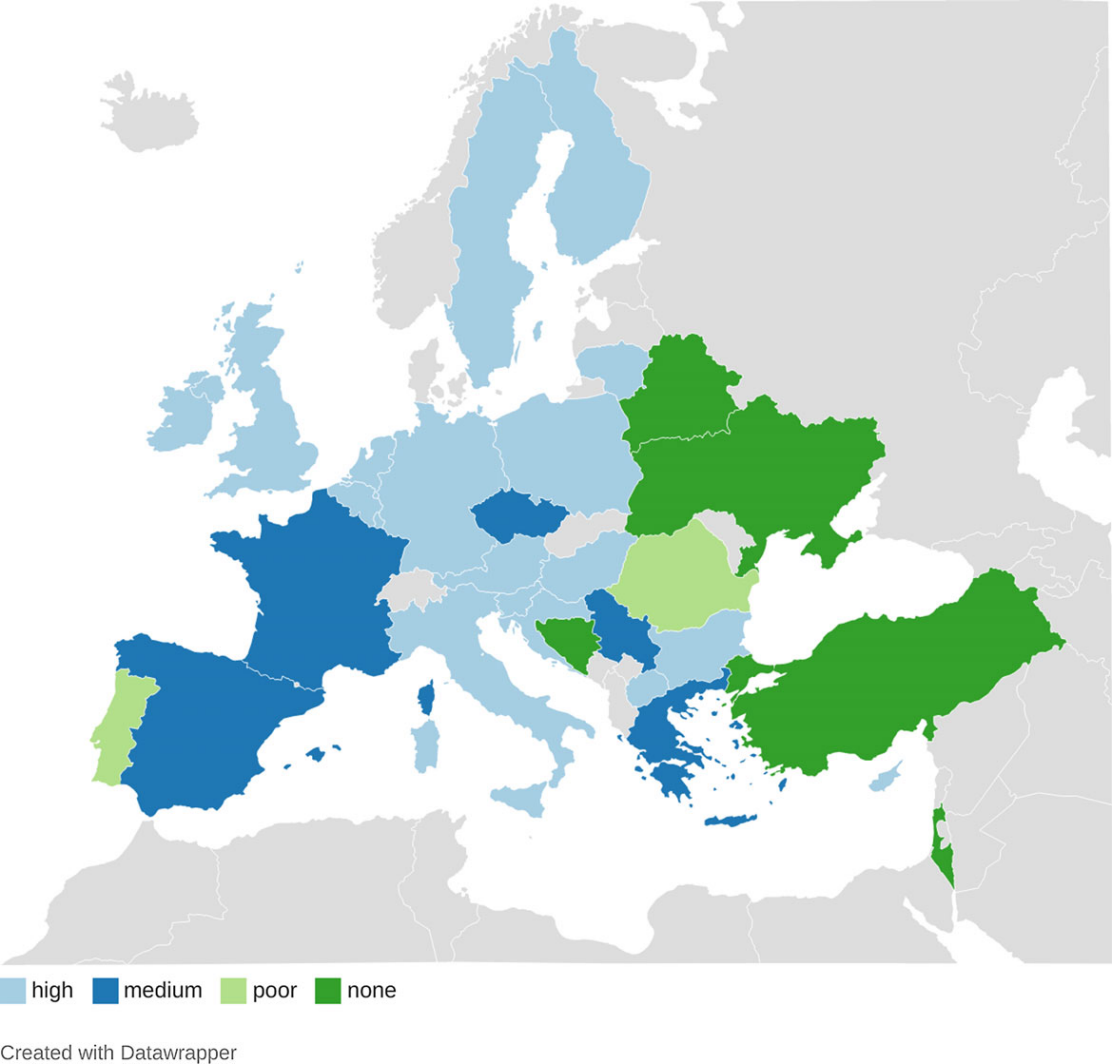
Participation of primary health care in medical care for COVID-19 patients living in long-term care facilities in Europe. Countries where primary health care (PHC) did not participate in providing medical care in long-term care facilities (LTCF). Poor: Countries where PHC rarely participated in providing medical care in LTCFs. Medium: Countries where PHC sometimes participated in providing medical care in LTCFs. High: Countries where PHC participated in providing medical care in LTCFs.

A retrospective descriptive study was performed in 30 countries (Figure 1). This article is part of the Eurodata study, which aims to describe the role of PHC during the COVID-19 pandemic in Europe (Ares-Blanco *et al*., 2023). In this study, the core research team was formed by six specialists in family medicine, preventive medicine, and public health, as well as a group of 45 national key informants from participating countries. The informants were invited through the European General Practice Research Network (EGPRN) and the European branch of the World Organization of Family Doctors (WONCA Europe). EGPRN is an organization of GPs and other health professionals involved in research in PHC and family medicine in Europe (EGPRN, 2022), and WONCA Europe is an academic and scientific society representing European GPs (Wonca, 2022). A presentation of the project took place at the EGPRN meeting in October 2021, and all the assistants were invited to participate. The main criteria for participation were either being a GP or having a background in GP, practicing in Europe during the pandemic and speaking English. All the key informants were health professionals and lead researchers in the different European countries represented in the study, mostly working in general practice, with the exception of the participants from Finland and Lithuania who were working in public health during the study period. Data were collected through an ad hoc semi-structured questionnaire intended to provide country-specific data about COVID-19 LTCF pathways from September 2020. The questionnaire was based on the clinical pathway described by the WHO (World Health Organization [WHO], 2020). Changes to the initial LTCF questionnaire were made by the core group before distributing it to all the key informants in October 2021. The questionnaire was circulated twice until a consensus was reached in November 2021, including a glossary with the definition of terms (Supplement 1). All the comments were included in a new version and all researchers provided feedback. An agreement was finally obtained in the second round. Three online meetings were organized to share the agreement and provide recommendations on how to collect the information from relevant and reliable official sources (Governmental guidelines—national and regional ones, scientific societies, medical consensus among clinicians). The sources are quoted in Supplement 2. The questionnaire was filled by one or two national key informants, and it was peer reviewed by a different national researcher before submitting it to the core group of researchers. They checked the national data to assure the data quality. The national information was collected between January and February 2022. Data analysis for qualitative variables was performed by organizing and transcribing the information from the questionnaires. The information was reviewed by the core group in March–April 2022. A national and international peer review was performed to assure the quality of the data. In cases where the information received was unclear, key informants were contacted to provide further details to complete the initial information.

Variables were grouped in five blocs: (i) SARS-CoV-2 testing, (ii) contact tracing, (iii) follow-up, (iv) additional testing, and (v) moderate and severe cases (Supplement 1). An international peer review of all the national data was performed by the core group. If there were differences in interpretations, they contacted the national key informant to clarify the description. They also homogenized the language to facilitate the interpretation of the data. All decisions regarding the language were agreed on with the key informants. Once that results were aggregated, we shared it with the key informants to confirm the findings, and all agreed with the results. A final consensus with the information of each country was achieved between the core group and the key informants in May 2022.

Medical care was defined as the initial care provided, COVID-19 testing, COVID-19 contact tracing, supervision of isolation, and medical care including physical examination, follow-up, and complementary tests.

The degree of PHC involvement in the LTCF COVID-19 patients’ clinical pathways in the different countries was defined as (Figure 1):

- PHC was the main provider of medical care (high).
- PHC was not the main provider of medical care; however, they deserved some complementary services or, in case of shortage of LTCF workforce, they became the main provider (medium).
- PHC did not deliver healthcare to LTCFs except for specific issues (poor).
- PHC was not involved in the medical care of these patients (none).

## Results

1. The role of PHC

PHC was involved in caring for LTCF COVID-19 patients in 26 out of the 30 European countries that participated in the study, either exclusively or in collaboration with other departments. The role of PHC was predominant in 17 countries (Figure 1). Furthermore, LTCFs received extra support from COVID-19 teams and infectious disease or internal medicine specialists in five countries.

1. The role of nurses in the LTCF

In Belarus, Belgium, Bulgaria, France, Israel, and Spain, nurses provided medical care alongside GPs or LTCF doctors. In France and Italy, nurses were responsible for performing antigenic tests and delivering the results to patients in Belarus, Bosnia and Herzegovina, Germany, Italy, Luxembourg, Spain, and Sweden. In Croatia and Spain, nurses worked with public health and PHC teams to conduct contact tracing.

1. Detection of cases

Isolation rooms for COVID-19 patients were available in all countries (red zones). However, in Austria and Ireland, LTCFs directly established red zones, while, in others, it was organized by public health or PHC. SARS-CoV-2 testing took place in LTCFs in 11 countries and was provided by PHC in seven countries, while contact tracing was mainly conducted by public health services (Table 2). When COVID-19 positive patients were detected in LTCFs, infected cases could be isolated in special areas (red zones) under the supervision of the staff (mostly nurses), except in Austria, where practices varied between facilities.

1. Care of COVID-19 patients

**Table 2.** Management of COVID-19 patients in long-term care facilities in European countries

Country	Initial medical care	Institution in charge of testing	Department doing contact tracing	Isolation supervision	Medical care and follow-up	Physical examination	Availability of X-ray	Availability of phlebotomy	Additional testing to end the isolation
Austria	GP/Hotline	PHC/Lab	Local government	Local Health Authorities	GP	GP	Hospital	Hospital	No
Belgium	LTCF staff/Coordinating GP	Coordinating GP	LTCF staff/Coordinating GP	LTCF staff/Coordinating GP	Coordinating GP	Coordinating GP/Patient’s GP	Ambulatory radiology service	Coordinating GP/Patient’s GP	No
Bulgaria	GP/Nurse/LTCF doctor	PHC/PH/Hospital/Hotline	PH/Regional Health Authorities	LTCF Staff	GP/A&E	LTCF A&E/Hospital	Hospital	Lab	No
Croatia	GP	PHC/Hospital/PH	LTCF nurses/PH	LTCF Staff/Civil Defense	GP/nurse	GP/A&E	COVID-19 hospital/A&E	COVID-19 Hospital/A&E	No
Cyprus	GP	Hospital/Lab	Ministry of Health	GP	GP	COVID-19 Outpatient clinic/Hospital	COVID-19 Outpatient clinic/Hospital	COVID-19 Outpatient clinic/Hospital	Yes
Czech Republic	GP	PHC/Mobile testing team/Lab	PH	LTCF Staff	GP	GP	PHC	PHC	Yes
Finland	App/GP	Lab/PHC	Municipalities/Hospital/PH	LTCF Staff	PHC	GP/A&E/PH	PHC/PH/A&E	PHC/PH/A&E	No
France	LTCF doctor or nurse/GP	LTCF doctors or nurses	National health insurance	LTCF Staff	GP/Nurses/LTCF doctor	GP/LTCF doctor	Hospital	LTCF	Yes
Germany	GP	Mobile testing center/PHC	PH	Nurses	GP	GP	Radiology clinic/Hospital	LTCF	Yes
Greece	LTCF doctor/PHC/Hotline	PHC/Hospital	PH/Civil Protection	LTCF Staff/PH	LTCF doctor/GP/Internist	LTCF doctor/GP/Internist	Radiology clinic/Hospital	LTCF	Yes, depending on the symptoms
Hungary	GP	LTCF staff	N/A	LTCF Staff	GP	GPLTCF staff	Radiology clinic/Hospital	LTCF	Yes
Ireland	PHC	National health service/PH	PH	LTCF Staff	LTCF staff/PHC	LTCF staff/PHC	A&E	A&E	No
Italy	LTCF GP/GP/Out of Hour Service	LTCF GP/PHC	LTCF Staff/PH	LTCF Staff	LTCF doctor/GP	LTCF doctor/GP	PHC/Hospital	LTCF	Yes
Lithuania	Hotline/GP	Hotline/PHC	PH	GPLTCF Staff	GP	GP or PHC nurse	Hospital	LTCF	No
Luxembourg	GP/Hotline/Out of hours	LTCF staff	LTCF staff/PH	N/A	LTCF GP/GP	LTCF GP/GP	Hospital	LTCF	No
Netherlands	LTCF doctor	PH	PH	LTCF doctor or nurse	LTCF doctor	LTCF doctor	Hospital	LTCF/Hospital	No
North Macedonia	PHC	PH	PH	LTCF Staff	GP/Infectious diseases specialist	COVID-19 Outpatient clinic/Hospital	Hospital	Lab	Yes
Poland	PHC	PHC/Hospital/Lab	Sanitary station	LTCF Staff	LTCF GP or nurse	GP	Hospital	LTCF	No
Romania	Ambulance/GP	Lab/PH	PH	PH/Police	GP	Ambulance	COVID-19 Outpatient clinic	COVID-19 Outpatient clinic	No
Serbia	PHC/PH/Hotline	PHC/PH	LTCF Staff/PH	LTCF GP or nurse	LTCF GP or nurse	LTCF GP /GP	COVID-19 Outpatient clinic	COVID-19 Outpatient clinic	Yes
Slovenia	LTCF GP	LTCF PHC team	PH	LTCF Staff	LTCF GP or nurse	LTCF GP	Hospital	LTCF	No*
Sweden	PHC	LTCF/PHC	Regional Infection Tracing Department	LTCF Staff	LTCF nurses/GP	GP	A&E	LTCF/PHC	No
Spain	LTCF doctor or nurse/GP	PHC/LTCF staff	LTCF doctor or nurse/PHC	LTCF Staff	LTCF doctor or nurse/GP	LTCF doctor	PHC/A&E	LTCF	No
United Kingdom	PH (Health protection team)	NHS England	LTCFS trace and test	N/A	Aligned primary care network/GP	Aligned primary care network/GP	PHC	PHC	Yes

Countries with no primary health care implication were Belarus, Bosnia and Herzegovina, Israel, Portugal, Turkey, Ukraine.

A&E = Accident and Emergency Department; GP = general practitioner; PH = public health; PHC = primary health care.

COVID-19 outpatient clinic: Primary care clinic that take care of COVID-19 ambulatory patients and it is run by primary care staff.

Aligned primary care network: Network of PHC practices that provide integrated and coordinated care in the community.

*The test was not needed but it was performed routinely before going out the COVID-19 zone in the LTCF.

In most countries, if patients presented with suspicious symptomatology, they contacted GPs for medical care; symptoms’ follow-up was mostly carried out by nurses from LTCFs and PHC. If physical examination was needed, GPs performed it in the LTCF. In Italy, they counted on the additional support of the *Unitá speciali di continuità assitenziale* (special continuity care units) for assisting patients under PHC direction. These units performed physical examinations, lung ultrasounds, and blood gas analysis (in some regions) in LTCFs and prescribed pharmacological therapies in collaboration with GPs.

No additional testing was performed in LTCFs. Chest X-rays could be requested by GPs in 12 countries and performed in hospitals or COVID-19-specific centers. For phlebotomies, a GP request was needed in 22 countries. When patients’ conditions worsened, they were referred to the hospital by LTCF staff or GPs.

## Discussion

This study describes the COVID-19 health care provided by PHC in LTCFs in 30 European countries. Nurses in LTCFs had an important role in the care of frail or old patients, testing, supervising isolation, and the follow-up of patients. PHC collaborated in the diagnosis and follow-up of COVID-19 in LTCFs. While many countries could perform SARS-CoV-2 testing and phlebotomies in LTCFs, chest X-rays were always taken in outpatient clinics.

In the European Union, the health care professionals involved in the care of LTCF residents are nurses, physiotherapists, and remunerated GPs at the national or regional level (Spasova *et al*., 2018). Before the pandemic, most of the Organization for Economic Co-operation and Development countries had developed some form of emergency preparedness protocols for their health systems but without special measures for LTCFs. However, after the pandemic, nearly all countries started including LTCFs in their plans (Rocard *et al*., 2021). After the first wave, the need for changes in regulations, funding, and strategies to care for these patients also became more obvious (Werner *et al*., 2020). In September 2020, SARS-CoV-2 testing was available in LTCFs of all the countries due to the implementation of the European Centre for Disease Prevention and Control (ECDC) guidelines (Adlhoch *et al.*, 2020). The COVID-19 pandemic has been confronted with a dynamic transformation of health, social, and economic structures (Haldane *et al.*, 2021). As is reflected in our results, two countries created specific organized networks to provide more integrated care: in Belgium, the *coördinerend en raadgevend arts (CRA)* was a specially trained consulting GP that coordinated medical care with the support of LTCF staff during the pandemic, and in the United Kingdom, the PHC network aligned with a group of practices that partnered with local communities at LTCFs to deliver care.

One review reported better nurse-to-patient ratios were associated with fewer cases, while nurse shortages were prone to cause COVID-19 outbreaks (Dykgraaf *et al.*, 2021). Proactive care from the nursing home staff, with regular communication and visits from their usual GP, seemed beneficial to LTCF residents (Sherlaw-Johnson *et al.*, 2018). Moreover, some countries (Estonia, Finland, Latvia, Luxembourg, Portugal, and Slovenia) promoted multidisciplinary teams to integrate PHC and LTCFs at the beginning of the pandemic (Rocard *et al*., 2021). France provided a new incentive to increase GP visits in LTCFs, whereas Italy and Luxembourg implemented medical care 24 hours. Our results highlight the role of nursing staff in the care of COVID-19 patients, as well as the role of GPs in performing physical examination, testing, and follow-ups, to guarantee continuity of care and attend those patients who would not benefit additionally from being admitted to a hospital (Miralles *et al.*, 2021). Indeed, nurses have played a key role in monitoring COVID-19 cases and contacting GPs in cases where patients’ conditions have worsened (British Geriatrics Society, 2020).

SARS-CoV-2 testing and phlebotomies were not available in LTCFs in all the countries, and some residents needed referrals to an outpatient or inpatient setting to access these tests. These results contrast with the recommendation from the WHO European Office to guarantee not only appropriate access to health care services in LTCFs but also an adequate provision of services in PHC (WHO, 2020). The results of our study show that hospital referral was recommended if severe COVID-19 was suspected; however, some other publications do not match our findings (Ouslander and Grabowski, 2020; Ryan, 2022; Shoaee *et al.*, 2022). In a report of six European countries, patients were not referred to the hospital if the incidence of pneumonia and COVID-19 cases had risen and intensive care units would provide patients *care according to age* policy, which implied that LTCF patients would not have access to all the treatments available at that time (Miralles et al., 2021). Furthermore, some pre-pandemic studies did not show benefits for frail elderly patients with pneumonia whether to be treated in intensive care unit or not, as life expectancy and comorbidities would not assure the patient’s recovery (Dosa, 2006; Loeb *et al.*, 2006; Tandan *et al.*, 2020).

### Strengths and limitations

**Figure 2.**
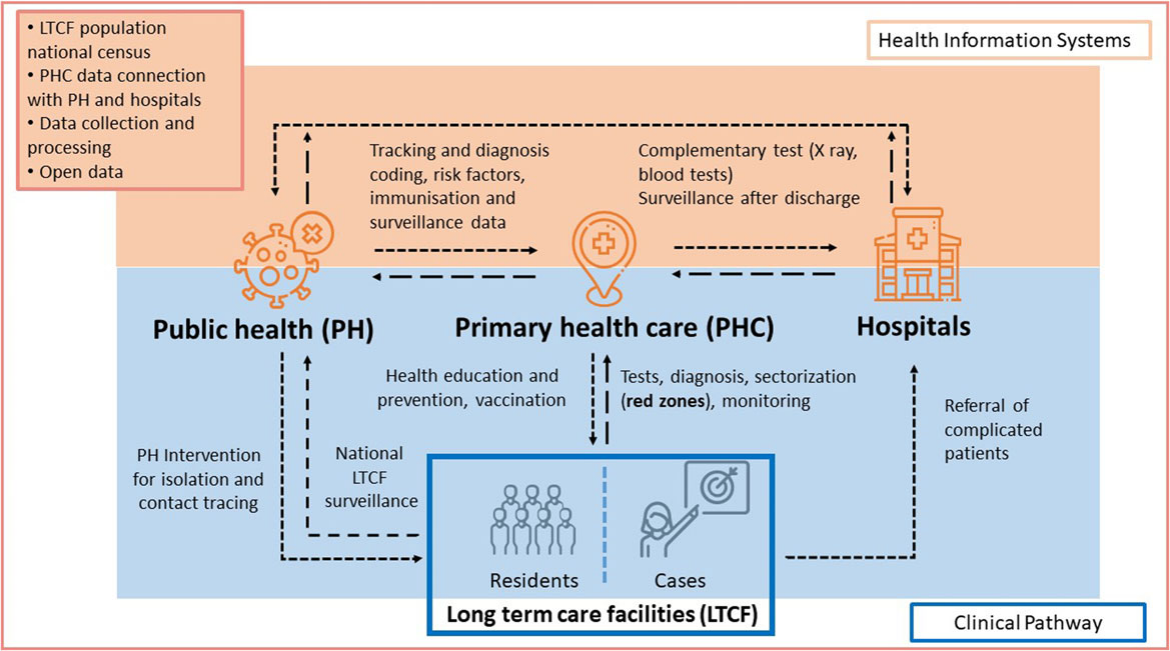
Roles and multilevel connections among primary health care, long-term care facilities, public health, and hospitals to care for the elderly during the pandemic.

To the best of our knowledge, this is the first detailed description of the role of PHC in the management of LTCF COVID-19 patients. This retrospective study spanning 30 European countries in the early stages of the COVID-19 pandemic has elucidated the interconnected roles and multilevel collaboration among PHC, LTCF, public health, and hospitals in the provision of care for elderly individuals during pandemics (Figure 2). A potential limitation could be the fact that all key informants were GPs. We did not consider to involve other kind of professionals who work in LTFC in the study (social workers, PHC nurses, or cases managers among others) as we wanted to collect the role of PHC in the COVID-19 clinical pathway in these facilities. Nonetheless, this bias was diminished by collecting information from publicly available official sources (Supplement 2) and also by the research background of key informants, GPs who belonged to the EGPRN of WONCA Europe.

As the organization and the number of LTCF beds vary across Europe, it is not possible to make a direct comparison of the pathways. However, a detailed description and juxtaposition might carry valuable information for future decision-makers and stakeholders of the different health care systems. As these are official data, we cannot contrast whether all recommendations were followed in the different regions of each country. Palliative care statistics of LTCF patients has not been collected. As the situation of palliative care was very different among the regions and depended on the pandemic peak waves, the information could vary widely inside each country.

The representation of data from two countries corresponds to a specific region and not to the entirety of the country. In the case of Sweden, the data were obtained from the Västra Götaland region, and for the United Kingdom, the corresponding information is specific to England.

### Implications for research and practice

Policies and further investment are needed to strengthen the coordination between PHC and LTCFs to improve patient-centered care (Figure 2). In addition, more research is required to examine the role of the different health care professionals involved in the care of LTCF residents, as well as qualitative research to explain the care preferences of the residents. Special care for LTCF residents should be established in future guidance when managing a pandemic.

Close collaboration between PHC professionals and nursing home staff is crucial for developing guidance on the management of COVID-19 to improve the comprehensive care of LTCF residents.

Further research is needed to understand the possible difficulties in separating the COVID-19 and non-COVID-19 residents and the capacity of LTCFs. Moreover, research related to all changes which took place in the management of care in LTCFs could provide relevant information for future pandemics. Additionally, it is necessary to describe the role of PHC in other diseases suffered by LTCF residents.

## Conclusion

The role of PHC and nurses in LTCFs during the COVID-19 pandemic has been decisive in many European countries, and LTCFs must be integrated in health care strategies when managing a pandemic. In the future, it is essential to value and promote the role of PHC professionals on pandemic management strategies, including coordination and integrated care within the health system, regarding LTCF health care provision.

## Supplementary material

For supplementary material accompanying this paper visit http://doi.org/10.1017/S1463423623000312. click here to view supplementary material
