# Supplementary material for: The role of primary health care in long-term care facilities during the COVID-19 pandemic in 30 European countries: a retrospective descriptive study (Eurodata study)
Source: Prim Health Care Res Dev. 2023 Oct 24;24:e60. doi: 10.1017/S1463423623000312 (PMC10594530; doi:10.1017/S1463423623000312)
Supplement: Supplementary file 1 [file phcsup.zip › S1463423623000312sup001.pdf]

SUPPLEMENT

Supplement 1: Final version of the questionnaire regarding the COVID-19 pathway in long-term care facilities in September 2020.

**1.SARS-CoV-2 testing**
If the patient has COVID-19 symptoms, who should he call to ask for medical care?
Which departments or institutions are in charge of RT-PCR?
Which departments or institutions are in charge of antigenic tests?
Where can patients get COVID-19 testing?
Is RT-PCR free of charge?
Is an antigenic test free of charge?
Can patients get a RT-PCR on their own without a prescription?
Can patients get an antigenic test without a prescription?
Who is testing patients who are immobile (elderly, bed ridden)?
Who is interpreting the result of the SARS-CoV-2 testing?
Who is in charge of giving the test result to the patient?
Other comments:
**2.Contact Tracing**
Which department is responsible for contact tracing?
Which channels are used to inform contact persons who are vulnerable? (Elderly, deaf patients, patients who don´t use a mobile phone, etc.)
Other comments:
**3.Follow-up**
How long is the length of the patient's isolation?
Who decides the lengths of the patient's isolation?
Who will supervise that the patient is doing the confinement correctly?
Who is giving medical care and follow-up to the patient's?
If the patient needs a physical exploration, who and where is the exam taking place?

If the patient's condition worsens, how will the patient communicate with their GP?
Other comments:
**4.Additional Testing**
If the patient needs an X-ray, who is requesting it and where will it happen?
If the patient needs a blood test, who is requesting it and where will it happen?
Do patients need a test at the end of the confinement to be allowed to end it?
Other comments:
**5.Moderate and severe cases**
In case of moderate or severe cases***, who is responsible of patient's care? When are patients referred to the hospital?
How do patients get transferred to the hospital (ambulance?) Who is responsible for ordering an ambulance?
Other comments:

Glossary (following the MeSH term in PubMed):

- **Primary care:** Care which provides integrated, accessible health care services provided by a GP or a primary care nurse in the context of family and community
- **A&E:** Accident & Emergency department or Emergency Department. Hospital department responsible for the administration and provision of immediate medical to the COVID-19 patient.
- **COVID-19 Telephone Hotline:** A direct communication system, usually telephone, established for instant contact. It is designed to provide only information about COVID-19 and assistance through trained personnel and is used for counseling and referrals.
- **Public health:** Branch of medicine concerned with the prevention and control of COVID-19 patients on the national, regional, or municipal level
- **GP:** General practitioner or family doctor. Doctors who are responsible for the provision of comprehensive and continuing care to every individual seeking medical care irrespective of age, sex and illness and they care for individuals in the context of their community (Wonca Europe definition, https://bit.ly/3D5HKfl)
- **Primary care nurse:** Nurses who provide care to patients of all age levels, and who focus their efforts on the health care needs of the entire family in the context of the community.

- **Public health doctor:** Doctor whose goal is to improve health and quality of life in a population or community through the prevention and treatment of diseases, the surveillance of cases and health indicators, and the promotion of healthy behaviors through public education and awareness.
- **Public health nurses**: Nurses whose goal is to improve health and quality of life in a population or community through the prevention and treatment of diseases, the surveillance of cases and health indicators, and the promotion of healthy behaviors through public education and awareness.
- **Social services:** The use of community resources, individual case work, or group work to promote the adaptive capacities of individuals in relation to their social and economic environments.
