# Supplementary material for: The role of primary health care in long-term care facilities during the COVID-19 pandemic in 30 European countries: a retrospective descriptive study (Eurodata study)
Source: Prim Health Care Res Dev. 2023 Oct 24;24:e60. doi: 10.1017/S1463423623000312 (PMC10594530; doi:10.1017/S1463423623000312)
Supplement: Supplementary file 1 [file phcsup.zip › S1463423623000312sup002.pdf]

Supplement 2: Sources of information (national or regional guidelines regarding clinical pathway of COVID-19 adult patients in primary health care)

COUNTRY	Sources of information
**Austria**	(*) Austria Federal Ministry of Social Affairs, Health, Care and Consumer Protection. Information about the Coronavirus. Coronavirus: specialist information. 2022.(Cited 14.4.2022) Available at: https://www.sozialministerium.at/Informationen-zum-Coronavirus/Coronavirus---Fachinformationen.html
**Belarus**	Links to Belarusian documents do not work from outside the country. However, the researcher can provide them on request:<br>Belarusian Government. Ministry of Health. Covid19 Information. Available at:<br>http://minzdrav.gov.by/upload/dadvfiles/law/%D0%BF%D1%80%D0%B8%D0%BA%D0%B0%D0%B7_%D0%9C%D0%97_11.11.2020_%E2%84%961195.pdf<br>http://minzdrav.gov.by/upload/dadvfiles/law/690.pdf<br>https://drive.google.com/file/d/1-0njxgMKBRVgsu4Cz3-3q5B_KlrINiER/view<br>https://drive.google.com/file/d/1063RYIclVfjaCQOZYZhr9nnTFpqamZY5/view<br>https://belmapo.by/normativnyie-dokumentyi-ministerstva-zdravooxraneniya-respubliki-belarus-po-infekczii-covid-19.html
**Belgium**	(*) Belgian Institute of Public Health. Sciensano: Coronavirus COVID-19. (Cited 29.5.2022). Available at: https://covid-19.sciensano.be/nl<br>(*) Agency of Health and care of the Flemish government. Newsletters and guidelines for nursing homes. (Cited 29.5.2022). Available at: https://www.zorg-en-gezondheid.be/corona-richtlijnen-voor-zorgprofessionals#ouderenzorg<br>(*) French speaking community: https://covid.aviq.be/fr/accueil-professionnels<br>(*) German speaking community: https://ostbelgiencorona.be/desktopdefault.aspx/tabid-7076/<br>(*) Brussels: https://coronavirus.brussels/nl/home/
**Bosnia and Herzegovina**	University of Banja Luka. Coronavirus Guidelines. May 2020. (Cited 22.4.2022). Available at: https://www.vladars.net/sr-SP-Cyrl/Vlada/Ministarstva/MZSZ/Documents/Prirucnik_za_lijecenje_.pdf<br><br>University of Banja Luka. Coronavirus Guidelines. February 2021. (Cited 22.4.2022). Available at: https://med.unibl.org/wp-content/uploads/2021/02/COVID-19-Smjernice-IV-izdanje.pdf<br><br>Sarajevo Canton. Ministry of Health. COVID19 Protocol. February 2021. (Cited 22.4.2022). Available at:<br>https://mz.ks.gov.ba/sites/mz.ks.gov.ba/files/protokol_za_lijecenje_covid_19.pdf<br><br>(*) Ministry of Health of Bosnia Herzogovina.COVID19 Protocol. February 2021. (Cited 22.4.2022). Available at:<br>https://covid19.fmoh.gov.ba/uploads/files/SMJERNICE%20ZA%20LIJE%C4%8CENJE%20OBOLJELIH%20OD%20COVID-19-f216cb5698d96c8000ff1a87b31f9154e185f30d.pdf
**Bulgaria**	(*) Bulgary. Ministry of Health. National COVID19 Information. April 2022. (Cited 22.4.2022). Available at: https://coronavirus.bg/bg/166

**Croatia**	Croatian Institute of Public Health. COVID19 Protocol. September 2020. (Cited 22.4.2022). Available at: https://www.hzjz.hr/wp-content/uploads/2020/03/Prioriteti_za_testiranje_ver_2_corr_01_09_2020.pdf<br><br>Croatian Institute of Public Health. COVID19 Protocol. March 2021. (Cited 22.4.2022). Available at: https://www.hzjz.hr/wp-content/uploads/2020/03/Postupanje-s-oboljelima-bliskim-kontaktima-oboljelih-i-prekid-izolacije-i-karantene-3.pdf<br><br>Ministry of Health of the Republic of Croatia. Guidelines for the management of coronavirus pain 2019 (COVID-19). Version 3 of 21. November 2021. (Cited 22.4.2022). Available at: https://www.hzjz.hr/wp-content/uploads/2021/11/Smjernice-za-lije%C4%8Denje-oboljelih-od-koronavirusne-bolesti-2019-COVID-19-verzija-3-od-21-listopada-2021.-godine.pdf
**Cyprus**	(*) Cyprus General Health System. April 2022. (Cited 22.4.2022). Available at: www.gesy.org.cy<br><br>(*) Cyprus. Ministry of Health Administration. April 2022. (Cited 22.4.2022). Available at: www.moh.gov.cy<br><br>Cyprus. New Coronavirus Disease (COVID19). Protocol March 2020 (Cited 22.4.2022). Available at: https://www.pio.gov.cy/coronavirus/eng
**Czech republic**	Bohemian General Medical Society. (Cited 22.4.2022). Available at: https://www.lkcr.cz/informace-262.html?do[loadData]=1&itemKey=cz_100266
**Finland**	Ministry of Health and Welfare. Finland. Public Coronavirus Guideline. September 2020. (Cited 22.4.2022): https://valtioneuvosto.fi/documents/1271139/38362681/Siikavirta_STM_THL_verkkosivuille_viet%C3%A4v%C3%A4ksi_240920.pdf/9999a89e-9338-b67e-bc24-14eb47480da2/Siikavirta_STM_THL_verkkosivuille_viet%C3%A4v%C3%A4ksi_240920.pdf?t=1600927765487<br><br>Finnish Institute of Health and Welfare. Transmission and protection coronavirus. Quarantine and isolation. 20.4. 2022 (Cited 24.4.2022). Available at: https://thl.fi/en/web/infectious-diseases-and-vaccinations/what-s-new/coronavirus-covid-19-latest-updates/transmission-and-protection-coronavirus/quarantine-and-isolation<br><br>Ministry of Health. Finland. Regional communicable disease control measures and preparedness of the service system. September 2020. (Cited 22.4.2022). Available at: https://stm.fi/documents/1271139/21429433/STM+ohjauskirje_alueelliset+tartuntatautien+torjunnan+toimenpiteet_10092020.pdf/0a031b18-662a-7792-9d74-d8a8b59a0507/STM+ohjauskirje_alueelliset+tartuntatautien+torjunnan+toimenpiteet_10092020.pdf?t=1600086597294<br><br>KELA, Social Security Institution. Kela reimbursement for coronavirus and antibody tests in private healthcare. April 2020. (Cited 22.4.2022). Available at: https://www.kela.fi/ajankohtaista-henkiloasiakkaat/-/asset_publisher/kg5xtoqDw6Wf/content/yksityisen-terveydenhuollon-koronavirustutkimuksista-ja-vasta-ainetesteista-saa-kela-korvauksen<br><br>Ministry of Health and Social Affairs of Finland and Finnish Institute of Health and Welfare. COVID 19 outbreak management implementation of a hybrid strategy - Preparing for a possible second wave – July 2020. (Cited 22.4.2022). Available at: https://stm.fi/documents/1271139/21475529/COVID+19+-epidemian+hallinnan+hybridistrategian+toteuttaminen+%E2%80%93+Mahdolliseen+toiseen+aaltoon+varautuminen.pdf/3c667955-2927-5067-8ca8-e51d79796d8e/COVID+19+-epidemian+hallinnan+hybridistrategian+toteuttaminen+%E2%80%93+Mahdolliseen+toiseen+aaltoon+varautuminen.pdf?t=1598343908408<br><br>Helsin University Hospital. Instructions for COVID19 Testing. September 2020. (Cited 22.4.2022). Available at: https://www.hus.fi/sites/default/files/2020-09/Koronabotti_ajanvaraus_koronatestiin_palvelun%20kuvaus.pdf

	Self-service Appointments. Uusimaa Region. Finland. September 2020. (Cited 22.4.2022). Available at: https://www.terveyskyla.fi/en/self-service-appointment-booking-for-coronavirus-testing-for-residents-of-uusimaa<br><br>Digifinland. Digital Platform for health and social services (Omaolo e-service channel for health care, social services and oral health care). Covid19 Training Course. Modul1. Coronavirus. September 2020. (Cited 22.4.2022). Available at: https://digifinland.fi/wp-content/uploads/2020/09/Nopeutettu_KA_Modul1_Korona190820.pdf<br><br>Ministry of Health and Social Affairs of Finland. Guideline of the ministry of social affairs and health: covid-19. Covid in primary social and health care services in the operation of basic health care services. March 2020. (Cited 22.4.2022). Available at: https://stm.fi/documents/1271139/21475529/Sosiaali-+ja+terveydenhuollon+perustason+palvelut,+sosiaali-+ja+terveysministeri%C3%B6n+p%C3%A4vitetty+ohje+16.4.2020.pdf/7a084845-fc42-0281-7191-67f8dac5c477/Sosiaali-+ja+terveydenhuollon+perustason+palvelut,+sosiaali-+ja+terveysministeri%C3%B6n+p%C3%A4vitetty+ohje+16.4.2020.pdf?t=1634902956611<br><br>Finnish Institute of Health and Welfare. Length of corona-related quarantine reduced to 10 days from 12.10. October 2020. (Cited 22.4.2022). Available at: https://thl.fi/fi/-/koronaan-liittyvan-karanteenin-pituus-lyhenee-10-paivaan-12.10.-alkaen<br><br>(*) Ministry of Health and Social Affairs of Finland. Social Emergency Services. (Cited 22.4.2022). Available at: https://stm.fi/sosiaalipaivystys<br><br>Ministry of Health and Social Affairs of Finland. Guideline of the ministry of social affairs and health: covid-19. Preparing for corona virus infections and customer needs Response to home services. June 2020. (Cited 22.4.2022). Available at: https://stm.fi/documents/1271139/21475529/Sosiaali-+ja+terveysministeri%C3%B6n+ohje_Koronavirustartuntoihin+varautuminen+ja+asiakkaiden+tarpeisiin+vastaaminen+kotiin+annettavissa+palveluissa_11.6..2020_p%C3%A4ivitetty/547b5022-ac2b-1475-9925-8c40b07e5025/Sosiaali-+ja+terveysministeri%C3%B6n+ohje_Koronavirustartuntoihin+varautuminen+ja+asiakkaiden+tarpeisiin+vastaaminen+kotiin+annettavissa+palveluissa_11.6..2020_p%C3%A4ivitetty.pdf?t=1634902956925<br><br>Ministry of Health and Social Affairs of Finland. Guideline of the ministry of social affairs and health: covid-19. Absence, quarantine and isolation decisions related to the COVID-19 pandemic. June 2020. (Cited 22.4.2022). Available at: https://stm.fi/documents/1271139/21475529/STM_Kuntainfo_2_2020_korona_karanteenip%C3%A4%C3%A4t%C3%B6kset_02.06.2020.pdf/ab09568c-251b-c3af-dae9-59a04efc1e91/STM_Kuntainfo_2_2020_korona_karanteenip%C3%A4%C3%A4t%C3%B6kset_02.06.2020.pdf?t=1634109984263
**France**	(*) Haute Autorite de Sante [High Authority of Health]. France. Coronavirus Information. Updated 7.4.2022. (Cited 22.4.2022). Available at: https://www.has-sante.fr/jcms/p_3165982/fr/coronavirus-covid-19<br><br>(*) Collèges de la Médecine General de France. Coronaclic: toutes les informations utiles pour les Médecins généralistes. Updated March 2022. (Cited 22.4.2022). Available at: https://lecmg.fr/coronaclic-8-mises-a-jour/<br><br>(*) Collège Nationale des Généralistes Enseignants. Les nouvelles recommandations HAS pour la prise en charge des patients suspectés de COVID en premier recours : une réelle avancée. Available at: https://www.cnge.fr/media/docs/cnge_site/cnge/201110_Communique_Recos_HAS_COVID_vsite.

	France Gouvernement. Décret n° 2021-13 du 8 janvier 2021 prévoyant l'application de dérogations relatives au bénéfice des indemnités journalières et de l'indemnité complémentaire prévue à l'article L. 1226-1 du code du travail ainsi qu'aux conditions de prise en charge par l'assurance maladie de certains frais de santé afin de lutter contre l'épidémie de Covid-19. (Cited 22.4.2022). Available at: https://www.legifrance.gouv.fr/loda/id/JORFTEXT000042895619/
**Germany**	(*) Ministry of Health. Germany. Information about Coronavirus Pandemic. Updated 14.4.2022. (Cited 22.4.2022). Available at: https://www.bundesgesundheitsministerium.de/coronavirus.html<br><br>(*) Robert Kock Institute. Germany. Infectious Diseases. Covid19 Information. Updated February 2022. (Cited 22.4.2022). Available at: https://www.rki.de/EN/Content/infections/epidemiology/outbreaks/COVID-19/COVID19.html<br><br>(*) Land Niedersachsen. Germany. Health and Social Welfare Ministry. Coronavirus Information. Updated 21.4.2022. (Cited 22.4.2022). Available at: https://www.niedersachsen.de/Coronavirus/<br><br>(*) Kassenärztliche Vereinigung Niedersachsen (KVN). Germany. Coronavirus Information. Updated 21.4.2022. (Cited 22.04.2022). Available at: https://www.kvn.de/Coronavirus.html
**Greece**	(*) National Public Health Organization. Coronavirus Information. Updated 21.4.2022. (Cited 22.04.2022). Available at: https://eody.gov.gr/en/covid-19/
**Hungary**	National Public Health Centre. Hungary. Procedures for the new coronavirus identified in 2020. Published 09 October 2020. (Cited 22.04.2022). Available at: https://www.nnk.gov.hu/index.php/koronavirus-tajekoztato/567-eljarasrend-a-2020-evben-azonositott-uj-koronavirussal-kapcsolatban<br><br>Ministry of Health. Hungary. Hungarian Coronavirus Management. November 2021. (Cited 22.04.2022). Available at: https://koronavirus.gov.hu/sites/default/files/sites/default/files/imce/magyar_koronavirus_kezikonyv_igazolt_covid-19_betegek_kezelese_cimu_fejezet_-_2021_november.pdf
**Ireland**	(*) Health Protection Surveillance Centre. Respiratory: Coronavirus Guidance. Updated 19.4.2022. (Cited 22.04.2022). Available at: https://www.hpsc.ie/a-z/respiratory/coronavirus/novelcoronavirus/guidance/newupdatedguidance/
**Israel**	(*) Director of Public Health of Israel. Coronavirus Medical Guidelines. Updated 19.4.2022. (Cited 22.04.2022). Available at: https://govextra.gov.il/media/17976/coronavirus_med_guidelines.pdf<br><br>(*) Director of Public Health of Israel. Coronavirus Medical Treatment Guidelines. Updated 19.4.2022. (Cited 22.04.2022). Available at: https://www.health.gov.il/Subjects/disease/corona/Documents/guide-treating-corona-patients.pdf<br><br>Association of Family Physicians of Israel. Guidelines of the Association of Family Physicians during the period of the global epidemic 19-CO. July 2020. (Cited 22.04.2022). Available at: https://cdn.mednet.co.il/2020/07/%D7%94%D7%A0%D7%97%D7%99%D7%95%D7%AA-%D7%90%D7%99%D7%92%D7%95%D7%93-%D7%A8%D7%95%D7%A4%D7%90%D7%99-%D7%94%D7%9E%D7%A9%D7%A4%D7%97%D7%94-%D7%91%D7%AA%D7%A7%D7%95%D7%A4%D7%AA-%D7%9E%D7%92%D7%A4%D7%AA-%D7%94%D7%A7%D7%95%D7%A8%D7%95%D7%A0%D7%94-.pdf

**Italy**	Ministry of Health of Italy. Collection of acts containing urgent measures for the containment and management of the epidemiological emergency by COVID-19 Collection of acts issued by the Ministry of Health. September 2020. (Cited 24.04.2022). Available at: https://www.gazzettaufficiale.it/eli/id/2020/09/07/20A04814/sg<br><br>(*) National Institute of Health of Italy. Coronavirus Information. Updated 21.4.2022. (Cited 24.04.2022). Available at: https://www.epicentro.iss.it/en/coronavirus/sars-cov-2-ipc
**Lithuania**	(*) Government Republic of Lithuania. Coronavirus Information. Updated 21.4.2022. (Cited 24.04.2022). Available at: https://koronastop.lrv.lt/
**Luxembourg**	(*) Luxembourg Government. Sanitary Measures. Covid19. Updated 21.4.2022. (Cited 24.04.2022). https://covid19.public.lu/en/sanitary-measures.html<br><br>(*) Luxembourg Government. Self-declaration of a COVID-19 infection / as a contact person. Updated 21.4.2022. (Cited 24.04.2022). Available at: https://covidtracing.public.lu/home
**Netherlands**	(*) National Institute for Public Health and the Environment. COVID-19. (Cited 29.05.2022). Available at https://www.rivm.nl/en/coronavirus-covid-19
**North of Macedonia**	North Macedonia. Institute of Public Health. Pathway for family doctors for testing and treatment of patients with COVID-19. March 2020. (Cited 24.04.2022). Available at: http://zdravstvo.gov.mk/wp-content/uploads/2020/04/Pateka-za-matichni-lekari-20.03.2020-f..pdf
**Poland**	(*) Poland Government. COVID19 General Information. April 2022. (Cited 1.5.2022). Available at: https://www.gov.pl/web/koronawirus/aktualne-zasady-i-ograniczenia<br><br>(*) College of Family Physicians in Poland. Rules of conduct regarding SARS-COV-2 infection recommended by KLRwP. April 2022. (Cited 1.5.2022). Available at: https://www.klrwp.pl/strona/644/koronawirus-calosc/pl<br><br>(*) Polish Society of Family Medicine. COVID19 documents regarding COVID-19, including the regulations, announcements of the Ministry of Health, recommendations regarding the diagnosis and treatment of COVID-19. March 2022. (Cited 1.5.2022). Available at: https://ptmr.info.pl/covid-19-akty-prawne/
**Portugal**	Portuguese General-Directorate for Health. Guideline 004/2020. Management to the patient with suspected or confirmed SARS-CoV-19 infection. Version 30/08/2020<br><br>Portuguese General-Directorate for Health. Guideline 010/2020: Management of the asymptomatic person with positive laboratory test. Version 15/04/2020<br><br>Portuguese General-Directorate for Health. Guideline 015/2020. COVID19: Contact tracing, version 15/04/2020<br><br>Recommendation 009/2020 Procedures for residential structures for elderly people, hospice care units, and other structures for elderly or foster care of children and young people. Version 07/04/2020

	Recommendation 015/2020 COVID-19: laboratory diagnosis, version from 23/04/2020
**Romania**	(*) Ministry of Health of Romania. Covid19 Information. Updated 22.4.2022. (Cited 24.4.2022). Available at: https://www.ms.ro/informatii-covid-19/<br><br>(*) Government of Romania. Covid19 Information. Updated 22.4.2022. (Cited 24.4.2022). Available at: https://gov.ro/ro/info-coronavirus-covid-19<br><br>(*) UN Refugee Agency-Romania. Covid19 Information. Updated 22.4.2022. (Cited 24.4.2022). Available at: https://www.unhcr.org/ro/covid-19<br><br>Romanian Government. COVID Briefing -19, Strategic Communication Group, 01 July, 13.00. July 2020. (Cited 24.4.2022). Available at: https://www.mai.gov.ro/informare-covid-19-grupul-de-comunicare-strategica<br><br>Romanian Government. ORDER No. 487of 23 March 2020approving the treatment protocol for SARS-Cov-2 virus infection. March 2020. (Cited 24.04.2022). Available at: https://www.anm.ro/_/ORDINE/OMS%20487%20din%2023%20martie%202020.pdf<br><br>Romanian Government. ORDER No 533 of 22 April 2021amending the Annex to Order of the Minister of Health No 487/2020 approving the treatment protocol for SARS-CoV-2 infection. April 2021. (Cited 24.04.2022). Available at: https://legislatie.just.ro/Public/DetaliiDocumentAfis/241318<br><br>Rumanian Government. COVID Information -19, Strategic Communication Group. July 2022. (Cited 22.4.2022). Available at: https://www.mai.gov.ro/informare-covid-19-grupul-de-comunicare-strategica<br><br>Rumanian Government. ORDER No. 487 *) of March 23, 2020 for the approval of the protocol for the treatment of SARS-Cov-2 virus infection. (Cited 22.4.2022). Available at: https://www.anm.ro/_/ORDINE/OMS%20487%20din%2023%20martie%202020.pdf<br><br>Rumanian Government. ORDER no. 533 of April 22, 2021 on amending the annex to the Order of the Minister of Health no. 487/2020 for the approval of the protocol for the treatment of SARS-CoV-2 virus infection. (Cited 22.4.2022). Available at: https://legislatie.just.ro/Public/DetaliiDocumentAfis/241318<br><br>Rumanian Government. ORDER no. 2,103 of October 12, 2021 on amending the annex to the Order of the Minister of Health no. 487/2020 for the approval of the protocol for the treatment of SARS-CoV-2 virus infection. (Cited 22.4.2022). Available at: http://legislatie.just.ro/Public/DetaliiDocument/247389<br><br>Romanian Government. Medication list and Covid-19 treatment protocol approved by WHO. 18_14.08.2020. (Cited 22.4.2022). Available at: https://www.anm.ro/_/ANUNTURI%20IMPORTANTE/lista%20medicamente%20conf%20protocol%20de%20tratatment%20al%20infectie%20cu%20COVID-19%20aprobata%20prin%20OMS%201418_14.08.2020.pdf
**Serbia**	Government of Serbia. Order prohibiting visits and restricting movement in the facilities of institutions for the accommodation of the elderly: 28 / 2020-14, 66 / 2020-17, 87 / 2020-34, 7 / 2021-54. (Cited 10.06.22). Available at: https://www.pravno-informacioni-sistem.rs/SlGlasnikPortal/eli/rep/sgrs/ministarstva/naredba/2020/28/1/reg

	Ministry of Health. Serbia. Professional-methodological instructions for control of the introduction and prevention of the spread of the new SARS-CoV-2 virus crown in the republic of Serbia. April 2020. (Cited 22.4.2022). Available at: http://demo.paragraf.rs/demo/combined/Old/t/t2020_04/PP_004_2020_002.htm<br><br>Government of Serbia. Article 6, paragraph 1 of the Law on Protection of the Population from Infectious Diseases ("Official Gazette of RS", No. 15/16) and Article 43, paragraph 1 of the Law on Government ("Official Gazette of RS", No. 55/05), 71/05 - correction, 101/07, 65/08, 16/11, 68/12 - US, 72/12, 7/14 - US, 44/14 and 30/18 - other law). The government brings DECISION declaring COVID-19 SARS-CoV-2 a contagious disease. (Cited 22.4.2022). Available at: https://www.pravno-informacioni-sistem.rs/SlGlasnikPortal/eli/rep/sgrs/vlada/odluka/2020/23/1/reg<br><br>Government of Serbia. Regulation on protection of the population from infectious diseases. "Official Gazette of RS", no. 15 of 25 February 2016, 68 of 10 May 2020, 136 of 13 November 2020. (Cited 22.4.2022). Available at: https://www.pravno-informacioni-sistem.rs/SlGlasnikPortal/eli/rep/sgrs/skupstina/zakon/2016/15/8/reg<br><br>Instructions on prevention and control measures spread of a new coronavirus (sars-cov-2) in health institutions. March 2020. (Cited 22.4.2022). Available at: https://www.zastitazdravlja.rs/docs/covid19/1MerePrevencijeUZdravstvenimUstanovama.pdf<br><br>Ministry of Health of Serbia. Algorithm / standard operating procedure March 12, 2020. Algorithm of activities for treatment of a person who is suspected of infection caused by new crown by Sars-Cov-2 virus. March 2020. (Cited 22.4.2022). Available at: https://www.zdravlje.gov.rs/vest/346428/algoritamstandardna-operativna-procedura.php<br><br>Serbia Government. Decree on the organization of the work of social protection institutions for the accommodation of beneficiaries and the organization of social protection for the provision of home accommodation services during a state of emergency. April 2020. (Cited 25.04.2022). Available at: https://www.propisi.net/uredba-o-organizovanju-rada-ustanova-socijalne-zastite-za-smestaj-korisnika-i-organizacija-socijalne-zastite-za-pruzanje-usluge-domskog-smestaja-za-vreme-vanrednog-stanja/<br><br>Ministry of Health of Serbia. Covid19 Measures. April 2020. (Cited 25.04.2022). Available at: https://www.minrzs.gov.rs/sites/default/files/vanredno-stanje/2020.04.06.%20Nalog%20003.pdf<br><br>(*) Ministry of Health of Serbia. General Information. Updated 24.4.2022 (Cited 25.4.2022). Available at: https://www.e-zdravlje.gov.rs/landing/
**Slovenia**	(*) Slovenian Public Health Institute. Covid19 Information. Updated 22.4.2022.(Cited 24.4.2022). Available at: https://www.nijz.si/sl/search/node/covid-19<br><br>Slovenian Public Health Institute. Covid-19 - new regulations on home quarantine. June 2021. (Cited 24.4.2022). Available at: https://www.nijz.si/en/covid-19-new-regulations-on-home-quarantine<br><br>(*) Ministry of Health of Republic of Slovenia. Covid19 Information. Updated 22.4.2022. (Cited 24.4.2022). Available at: https://www.gov.si/teme/koronavirus-sars-cov-2/
**Spain**	Ministry of Health. Spain. Primary Care COVID19 Management. July 2020. (Cited 24.4.2022). Available at: https://www.sanidad.gob.es/profesionales/saludPublica/ccayes/alertasActual/nCov/documentos/Manejo_primaria.pdf<br><br>Ministry of Health. Spain. Home management of end-of-life patients requiring palliative sedation in the context of the context of the COVID-19 pandemic. July 2020. (Cited 24.4.2022). Available at:

	https://www.sanidad.gob.es/profesionales/saludPublica/ccayes/alertasActual/nCov/documentos/18_06MANEJOENDOMICILIODEPACIENTESREQUIERENSEDACION.pdf<br><br>Ministry of Health. Spanish Agency for Medication and Health Products. Available treatments subject to special access conditions for the management of SARS-CoV-2 respiratory infection. July 2020. (Cited 24.4.2022). Available at: https://www.aemps.gob.es/la-aemps/ultima-informacion-de-la-aemps-acerca-del-covid%E2%80%9119/tratamientos-disponibles-para-el-manejo-de-la-infeccion-respiratoria-por-sars-cov-2/?lang=en<br><br>Ministry of Health. Spain. Emergency Management of COVID19 Patients. June 2020. (Cited 24.4.2022). Available at: https://www.sanidad.gob.es/profesionales/saludPublica/ccayes/alertasActual/nCov/documentos/Manejo_urgencias_pacientes_con_COVID-19.pdf<br><br>Ministry of Health. Spain. Interpretation of Diagnostic Tests of SARS Cov2. April 2020. (Cited 24.4.2022). Available at: https://www.sanidad.gob.es/profesionales/saludPublica/ccayes/alertasActual/nCov/documentos/INTERPRETACION_DE_LAS_PRUEBAS.pdf<br>Ministry of Health. Spain. Management of the pregnant woman and newborn with COVID-19. June 2020. (Cited 24.4.2022). Available at: https://www.sanidad.gob.es/profesionales/saludPublica/ccayes/alertasActual/nCov/documentos/Documento_manejo_embarazo_recien_nacido.pdf
**Sweden**	(*) Public Health Agency of Sweden. COVID19 General Information. Updated 22.4.2022. (Cited 24.4.2022). Available at: https://www.folkhalsomyndigheten.se/<br><br>(*) Västra Region. Sweden. Healthcare provider website Information on COVID19. Updated 22.4.2022. (Cited 24.4.2022). Available at: https://www.vgregion.se/halsa-och-vard/vardgivarwebben/vardriktlinjer/covid-19/kommun/<br><br>(*) Public Health Agency of Sweden. Västra Region. COVID19 General Information. Updated 22.4.2022. (Cited 24.4.2022). Available at: https://www.vgregion.se/halsa-och-vard/vardgivarwebben/vardriktlinjer/smittskydd-vastra-gotaland/
**Turkey**	General Directorate of Public Health. Turkey. COVID19 Patient Monitoring at Home. March 2020. (Cited 1.5.2022). Available at: https://hsgm.saglik.gov.tr/depo/covid19/Ingilizce/Algoritmalar/COVID19-_Evde_Hasta_Izlemi_ENG.pdf<br><br>General Directorate of Public Health. Turkey. Guide on possible Covid-19 case inquiry for outpatients. March 2020 (Cited 1.5.2022). Available at: https://hsgm.saglik.gov.tr/depo/covid19/Ingilizce/Algoritmalar/COVID19-VAKA-SORGULAMA-KILAVUZU-A4_1_ENG.pdf<br><br>General Directorate of Public Health. Turkey. Discharge and isolation rules in Covid-19 patients. March 2020 (Cited 1.5.2022). Available at: https://hsgm.saglik.gov.tr/depo/covid19/Ingilizce/Algoritmalar/COVID19-TaburculukveIzolasyonKurallari-28032020.pdf_ENG.pdf<br><br>General Directorate of Public Health. Turkey. COVID-19 (SARS-cov-2 INFECTION) (Study of Scientific Board) general information, epidemiology, and diagnosis. June 2020. (Cited 1.5.2022). Available at: https://hsgm.saglik.gov.tr/depo/covid19/Ingilizce/Rehber/COVID-19_Rehberi__Genel_bilgiler_epidemiyoloji_ve_tani_8.06.2020_eng.pdf<br><br>General Directorate of Public Health. Turkey. COVID-19 (SARS-cov-2 INFECTION) (Study of Scientific Board) general information, epidemiology, and diagnosis. May 2020. (Cited 1.5.2022). Available at: https://hsgm.saglik.gov.tr/depo/birimler/goc_sagligi/covid19/rehber/COVID-19_Rehberi20200414_eng_v4_002_14.05.2020.pdf<br><br>General Directorate of Public Health. Turkey. COVID-19 (SARS-cov-2 INFECTION) Clinical Algorithm Management. Mach 2020. (Cited 1.5.2022). Available at: https://hsgm.saglik.gov.tr/depo/covid19/Ingilizce/Algoritmalar/COVID19-PLKACILHASTAYONETIMI_ENG.pdf

**Ukraine**	Order of the Ministry of Health of Ukraine dated 20.09.2021 № 1979 "On amendments to the protocol" Provision of medical care for the treatment of coronavirus disease (COVID-19) ". September 2021. (Cited 24.4.2022). Available at: https://moz.gov.ua/article/ministry-mandates/nakaz-moz-ukraini-vid-20092021--1979-pro-vnesennja-zmin-do-protokolu--nadannja-medichnoi-dopomogi-dlja-likuvannja--koronavirusnoi-hvorobi-covid-19
**United Kingdom**	United Kingdom Government. Coronavirus and self-isolation after testing positive in England. April 2021. (Cited 1.5.2022). Available at: https://www.ons.gov.uk/releases/coronavirusandselfisolationaftertestingpositiveinengland12aprilto16april2021<br><br>United Kingdom Government. COVID-19: guidance for households with possible coronavirus infection. September 2020. (Cited 1.5.2022). Available at: https://www.gov.uk/government/publications/covid-19-stay-at-home-guidance

* National Health Institution link where the National COVID-19 protocols were available. Nevertheless, those from 2020 are not available nowadays on the web. The authors can provide them on request.
